# Perineuronal nets are under the control of type-5 metabotropic glutamate receptors in the developing somatosensory cortex

**DOI:** 10.1038/s41398-021-01210-3

**Published:** 2021-02-18

**Authors:** Giada Mascio, Domenico Bucci, Serena Notartomaso, Francesca Liberatore, Nico Antenucci, Pamela Scarselli, Tiziana Imbriglio, Stefano Caruso, Roberto Gradini, Milena Cannella, Luisa Di Menna, Valeria Bruno, Giuseppe Battaglia, Ferdinando Nicoletti

**Affiliations:** 1 IRCCS Neuromed, Pozzilli, Italy; 2 Department of Physiology and Pharmacology, Sapienza University, Rome, Italy; 3 Department of Experimental Medicine, Sapienza University, Rome, Italy

**Keywords:** Neuroscience, Physiology

## Abstract

mGlu5 metabotropic glutamate receptors are highly functional in the early postnatal life, and regulate developmental plasticity of parvalbumin-positive (PV^+^) interneurons in the cerebral cortex. PV^+^ cells are enwrapped by perineuronal nets (PNNs) at the closure of critical windows of cortical plasticity. Changes in PNNs have been associated with neurodevelopmental disorders. We found that the number of *Wisteria Fluoribunda Agglutinin* (WFA)^+^ PNNs and the density of WFA^+^/PV^+^ cells were largely increased in the somatosensory cortex of mGlu5^−/−^ mice at PND16. An increased WFA^+^ PNN density was also observed after pharmacological blockade of mGlu5 receptors in the first two postnatal weeks. The number of WFA^+^ PNNs in mGlu5^−/−^ mice was close to a plateau at PND16, whereas continued to increase in wild-type mice, and there was no difference between the two genotypes at PND21 and PND60. mGlu5^−/−^ mice at PND16 showed increases in the transcripts of genes involved in PNN formation and a reduced expression and activity of type-9 matrix metalloproteinase in the somatosensory cortex suggesting that mGlu5 receptors control both PNN formation and degradation. Finally, unilateral whisker stimulation from PND9 to PND16 enhanced WFA^+^ PNN density in the contralateral somatosensory cortex only in mGlu5^+/+^ mice, whereas whisker trimming from PND9 to PND16 reduced WFA^+^ PNN density exclusively in mGlu5^−/−^ mice, suggesting that mGlu5 receptors shape the PNN response to sensory experience. These findings disclose a novel undescribed mechanism of PNN regulation, and lay the groundwork for the study of mGlu5 receptors and PNNs in neurodevelopmental disorders.

## Introduction

Postnatal maturation of PV^+^ GABAergic interneurons is associated with the progressive build-up of perineuronal nets (PNNs), which represent a condensed form of the extracellular matrix surrounding the cell soma and proximal dendrites^1^. PNNs are enriched in chondroitin sulfate proteoglycans (CSPGs), such as aggrecan, versican, neurocan and brevican, which are linked to hyaluronic acid and tenascin-R. PNNs restrain the plasticity of PV^+^ interneurons, and their deposition coincides with the closure of “critical periods” of cortical plasticity^2,3^. Formation of PNNs dampens plasticity and helps consolidate circuitry around PV^+^ cells^4^. Genetic deletion of PNN-forming enzymes, such as chondroitin 6-O sulfotansferase-1 and N-acetylgalactosamine transferase-1, causes abnormal PV^+^cell maturation and cortical plasticity^5,6^. Alterations in the number and composition of PNNs have been found in the brain of individual affected by schizophrenia^7–10^, suggesting that mechanisms that regulate PNN formation and/or degradation can be targeted by therapeutic intervention. Matrix metalloproteinase (MMP-9) cleaves aggrecans, thereby influencing the stability of PNNs^11,12^. MMP-9 is highly expressed during early postnatal development and has been associated to neurodevelopmental disorders^13,14^. Several other molecules are critical for the formation, integrity, and function of PNNs, such as the hyaluronic acid associated proteins (Hapln1 and Tn‐R), and CSPGs^15^.

How glutamatergic transmission regulates PNN turnover across postnatal development is largely unknown. Glutamate activates both ionotropic (AMPA, NMDA, and kainate) and metabotropic (mGlu) receptors. There are eight mGlu subtypes, of which mGlu1 and -5 are coupled to G_q/11_, whereas all other subtypes are coupled to G_i/o_^16^. Studies carried out in organotypic brain cultures led to the conclusion that the glutamatergic system is not essential for PNN development. However, these studies used weak and non selective glutamate receptor antagonists, such as kynurenic acid for ionotropic glutamate receptors, and α-methyl-4-carboxyphenylglycine for mGlu receptors^17^. More recent in vivo data raise the possibility that PNN formation can be regulated by glutamate receptors. Genetic deletion of the NMDA receptor subunit GluN2A in mice delays PNN maturation in the anterior cingulate cortex^18^, whereas mice lacking mGlu3 receptors show an increased PNN density in the somatosensory cortex^19^.

Here, we focused on mGlu5 receptors, which are highly expressed and functional in the early postnatal life^20–23^. Postnatal ablation of mGlu5 receptors from PV^+^ neurons decreases inhibitory currents, impairs rhythmic cortical oscillatory activity, and induces alterations in sensory-motor gating, learning and memory processes and social recognition^24^. In addition, mice lacking mGlu5 receptors show a reduced expression of PV and other markers of GABAergic interneurons in the cerebral cortex and hippocampus^25^. These findings suggest a role for mGlu5 receptors in the development of PV^+^ neurons. Of note, mGlu5 receptors physically and functionally interact with NMDA receptors^26,27^, which are highly expressed and constitutively active in PV^+^ neurons^28^.

We examined whether the developmental trajectory of PNNs is under the control of mGlu5 receptors focusing on the somatosensory cortex because: (i) a large body of information is present on the expression, laminar distribution, and function of PNNs in the mouse somatosensory cortex^29–33^; and (ii) mGlu5 receptors regulate the formation of somatosensory maps during early postnatal development^34^.

## Materials and methods

### Animals

All mice were kept under controlled conditions (ambient temperature, 22 °C; humidity, 40%) on a 12 h light/dark cycle with food and water *ad libitum*. All experiments were carried out according to the European (86/609/EEC) and Italian (D. Lgs. 26/2014) guidelines of animal care and approved by the Ethical Committee of Neuromed Institute (Pozzilli, Italy) and funded by the Italian Ministry of Health. Heterozygous B6;129-*Grm5*^*tm1Rod*^*/*J mice were obtained from Jackson Laboratories (Bar Harbor, ME; Stock number 003558). mGlu5^−/−^ mice and mGlu5^+/+^ littermates were generated by heterozygous breeding. Mice from 2 litters were combined in most of experiments with no randomization. Mouse pups of both sexes were used at postnatal day (PND) 9, 16, 21. Only male mice were used at PND60; the day of birth was designed as PND0.

### Experimental design

Pilot experiments showed a good reliability of our PNN counting methods with a S.D. <10%. Thus, we chose sample sizes ranging from 3 to 6 invidual animals for PNN counting. For Western blot and transcipt analysis, sample sizes were 3–5, and 5–6, respectively. Separate groups of mGlu5^−/−^ and mGlu5^+/+^ mice were used for the assessment of: *Wisteria Floribunda Agglutinin* (WFA)^+^ PNN density at PND9, PND16, PND21, and PND60 (*n* = 4); WFA^+^/PV^+^ and PV^+^ cell density at PND16 and PND60 (*n* = 3–4); stereological counting of WFA^+^ PNNs at PND16 (*n* = 3–4) and PND60 (*n* = 4–5); immunoblot analysis of PV at PND16 and MMP-9 at PND16 and PND60 (*n* = 3–5); zymographic analysis of MMP-9 activity at PND16 (*n* = 4) and gene expression analysis at PND16 and PND60 (*n* = 5–6). Two groups of mGlu5^−/−^ and mGlu5^+/+^ (*n* = 4–5) mice were used for whisker stimulation, and two additional groups (*n* = 4) for sensory deprivation experiments (see below). All these mice were used for the assessment of WFA^+^, WFA^+^/PV^+^, and PV^+^ cell density. Two groups of control mice were treated i.p. with either saline (*n* = 3) or the mGlu5 receptor negative allosteric modulator (NAM), 3-((2-methyl-4-thiazolyl)ethynyl)pyridine (MTEP) (3 mg/kg) (*n* = 4), once a day from PND7 to PND14 and killed at PND16 for the assessment of WFA^+^, WFA^+^/PV^+^, and PV^+^ cell density. The same treatment was performed in two additional groups of control mice, in which, however, either saline (*n* = 4) or MTEP (*n* = 4) were injected daily from PND16 to PND21 (mice were killed 1 h after the last injection).

### Repetitive unilateral whisker stimulation

Left whisker stimulation was performed from PND9 to PND16. The whiskers were gently stimulated with a thin brush for 120 s every 5 s (2 sweeps, back and forth, across the entire whisker extention). Stimulation was repeated 4 times a day every 2 h, whithout touching the skin.

### Sensory deprivation

For the induction of unilateral sensory deprivation, all whiskers of the left side were trimmed every other day from PND9 to PND16 by means of surgical scissors. Control mice were handled every other day without whisker trimming^32^.

### Immunofluorescent staining of WFA^+^ PNNs

(See Supplementary). In brief, 30 μm brain sections were stained with WFA, a lectin that binds N-acetylgalactosamines-β1 residues of PNN. Fluorescent staining was performed using biotin-conjugate WFA (1:1000; #L1516, Sigma-Aldrich, St. Louis, MO) and secondary antibodies (Streptavidin Alexa Fluor 488, 1:200, #S32354, Invitrogen, Carsband, CA). We performed double staining for WFA and PV (monoclonal rabbit anti-PV antibodies; 1:1000, Swant, Switzerland; #PV27) using secondary Alexa Fluor 488 antibodies (1:200) and donkey anti rabbit Cy3 secondary antibodies (1:200, #711-165-152, Jackson ImmunoResearch, Cambridge, UK). Sections were examined with a ZEISS 780 confocal laser scanning microscope, a Zeiss Carl Apotome2 microscopy (Zeiss, Gottingen, Germany) and a Thunder Imaging System by Leica Microsystem (Wetzlar, Germany) and processed with ZEN software and/or LAS X software. Cell counting was performed unilaterally in three coronal sections (+0.98, +0.02, and −1.06 mm from Bregma), at 10x magnification from an observer who was aware of the treatment.

### Stereological counting of WFA^+^ PNNs in the somatosensory cortex

(See Supplementary). The absolute number of WFA^+^ PNNs in the whole extension of the somatosensory cortex and the medial portion of prefrontal cortex (cingulate cortex, area 1; prelimbic cortex; and infralimbic cortex) was assessed by an observer who was unaware of the mouse genotype by stereological technique and optical fractionator using a Zeiss Axio Imager M1 microscope equipped with a motorized stage, a focus control system (Zeta axis), and a digital video camera. The software Image-Pro Plus 6.2 for Windows (Media Cybernetics, Rockville, MD, USA) equipped with a Macro was used for the analysis of digital images. The Macro was obtained by Imagine and Computer (Milan, Italy). The total number of WFA^+^ PNNs was computed according to the formula: *N* = Σ(*n*) × 1/SSF × 1/ASF × 1/TSF, where n is the number of cells counted on each disector; SSF (fraction of sections sampled) is the number of regularly spaced sections used for counts divided by the total number of sections across the areas; ASF (area sampling frequency) is the disector area divided by the area between disectors (disector area × disector number/region area); and TSF (thickness sampling frequency) is the disector thickness divided by the section thickness. The Cavalieri estimator method was used to evaluate the volume of the somatosensory cortex.

### Immunoblot analysis

(See Supplementary). Primary antibodies: rabbit polyclonal anti-MMP-9 (Abcam, #ab38898; 1:1000), rabbit polyclonal anti-PV (Swant, Switzerland, #PV27, 1:1000) and mouse monoclonal anti-β-actin (Sigma-Aldrich, St. Louis, MO, #A5316; 1:50.000).

### Zymography analysis of MMP-9 activity

(See Supplementary). Proteins were separated under non denaturing conditions in 10% polyacrilamide gel containing gelatin (3 mg/ml). Gels were stained with Coomassie Blue 0.5% for 1 h and destained by acetic acid in methanol and H_2_O (50:10:40) for 1 h to visualize bands with gelatinolytic activity.

### Gene expression analysis

(See Supplementary). We measured the transcripts of genes involved in PNN formation and degradation by quantitative PCR. The sequence of all primers is reported in the Supplementary Materials.

### Statistical analysis

In all graphs, values are means + SEM. Statistical analysis was performed by Student’s *t* test and Two Way ANOVA followed by Bonferroni post-hoc test.

## Results

### Developmental profile of PNNs in the somatosensory cortex of wild-type and mGlu5 receptor knockout mice

In the mouse somatosensory cortex, PNNs become detectable at PND7-10, and continue to develop until 5 weeks of age^29,32,35^. We examined the expression of PNNs in mGlu5^+/+^ and mGlu5^−/−^ mice at PND9, PND16, PND21 and PND60.

**Fig. 1:**
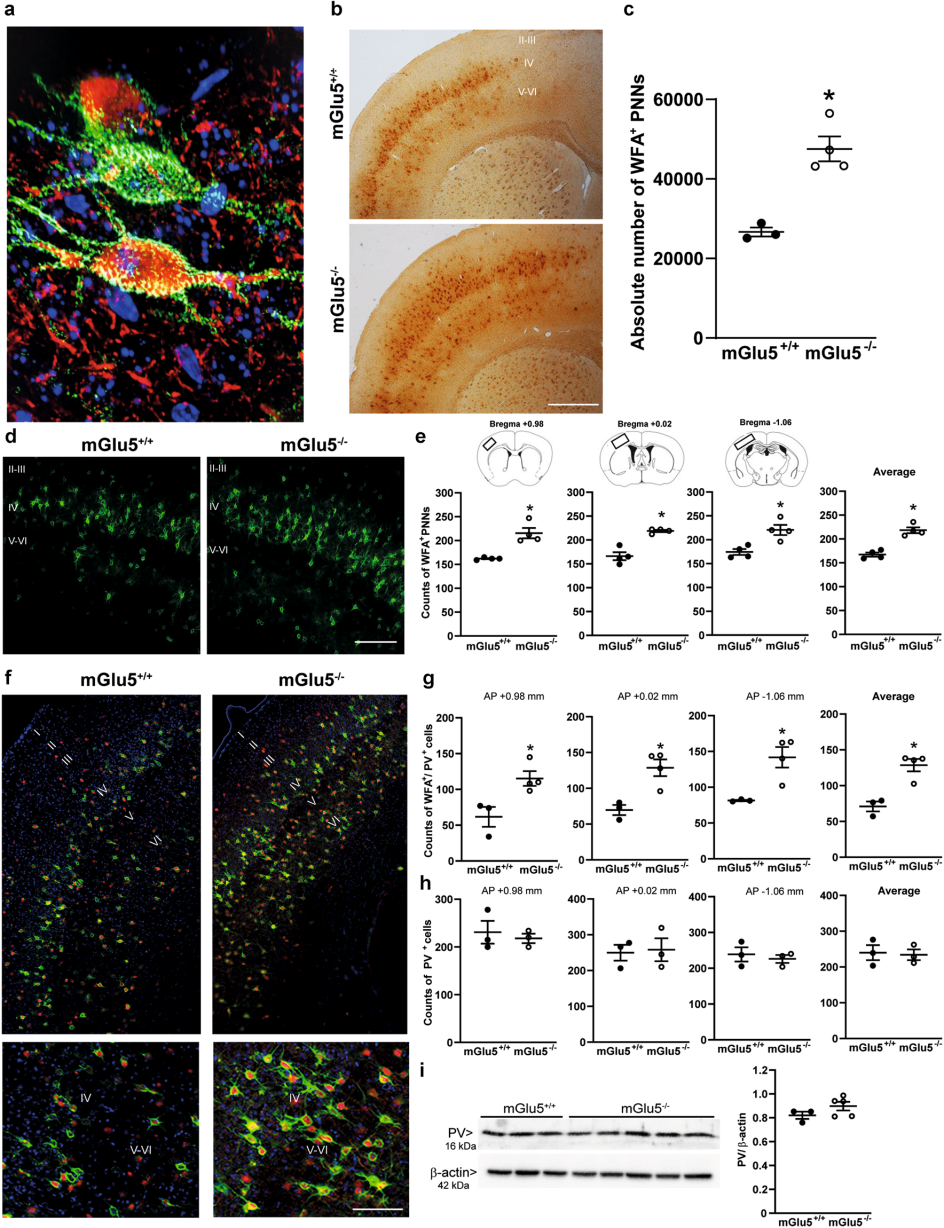
Up-regulation of PNNs in the somatosensory cortex of mGlu5^−/−^ mice at PND16. **a** Thunder high resolution image showing WFA^+^ PNNs (green) and PV^+^ neurons (red) (counterstained with DAPI). **b**–**c** Stereological counting of WFA^+^ PNNs in the somatosensory cortex (**b**, **c**). Scale bar = 100 μm (**b**). In **c** *p < 0.01 vs. mGlu5^+/+^ (Student’s *t* test; t5 = −5.475). **d**, **e** Fluorescent WFA staining and assessment of WFA^+^ PNN density; **d** scale bar = 50 μm. **e** *statistically significant vs. the corresponding mGlu5^+/+^ sections (Student’s *t* test); t6 = −4.92, *p* < 0.01 (AP + 0.98 mm); t6 = −6.055, *p* < 0.01 (AP + 0.02 mm); t6 = −3.75, *p* = 0.01 (AP – 1.06 mm); t6 = −4.906, *p* = 0.003 (average counts). **f**–**h** WFA (green) and PV (red) staining and assessment of WFA^+^/PV^+^ and PV^+^ cell density. **f** Thunder high resolution tailing images counterstained with DAPI. Scale bar = 25 μm. **g** *Statistically significant vs. the corresponding mGlu5^+/+^ sections (Student’s *t* test); t5 = −3.192, *p* = 0.024 (AP + 0.98 mm); t5 = −3.928, *p* = 0.011 (AP + 0.02 mm); t5 = −3.57, *p* = 0.016 (AP – 1.06 mm); t5 = −4.891, *p* = 0.005 (average counts). **i** Immunoblot analysis of PV. Immunostaining in **d**, **e** and in **f**–**h** was performed in independent groups of mice.

At PND9, WFA^+^ PNNs were present at low density in layer IV of the somatosensory cortex, and there was no difference between mGlu5^+/+^ and mGlu5^−/−^ mice (Supplementary Fig. 1). In contrast, a large difference between the two genotypes was found at PND16, when PNNs showed a granular structure and enwrapped both PV^+^ and PV^−^ neurons. There were also neurons in which PV did not colocalize with WFA (Fig. 1a). The absolute number of WFA^+^ PNNs was almost doubled in PND16 mGlu5^−/−^ mice as shown by unbiased stereological counting in the whole extension of the somatosensory cortex (Fig. 1b, c). We also measured the absolute number of WFA^+^ PNNs in the medial portion of the prefrontal cortex containing area 1 of the cingulate cortex, prelimbic and infralimbic cortex. The number of WFA^+^ PNNs was very low in these regions at PND16, and did not differ between the two genotypes (Supplementary Fig. 2a–d).

Data obtained with stereological cell counting were confirmed by measuring the density of WFA^+^ PNNs after immunofluorescent staining in three anatomical sections of the somatosensory cortex (+0.98, +0.02, and −1.06 mm from bregma) and their average number (Fig. 1d, e). The density of WFA^+^ PNNs enwrapping PV^+^ interneurons was also greater in mGlu5^−/−^ mice (Fig. 1f, g) whereas the density of PV^+^ cells (Fig. 1h) and PV protein levels (Fig. 1i) did not change. At PND16, ~40% of the total WFA^+^ PNNs enwrapped PV^+^ cells in the somatosensory cortex of wild-type mice (compare average counts of Fig. 1e, g). The large increase in the density of WFA^+^/PV^+^ cells found in mGlu5^−/−^ mice at PND16 (Fig. 1g) suggests that the receptor mainly regulates PNN formation/degradation around PV^+^ neurons.

**Fig. 2:**
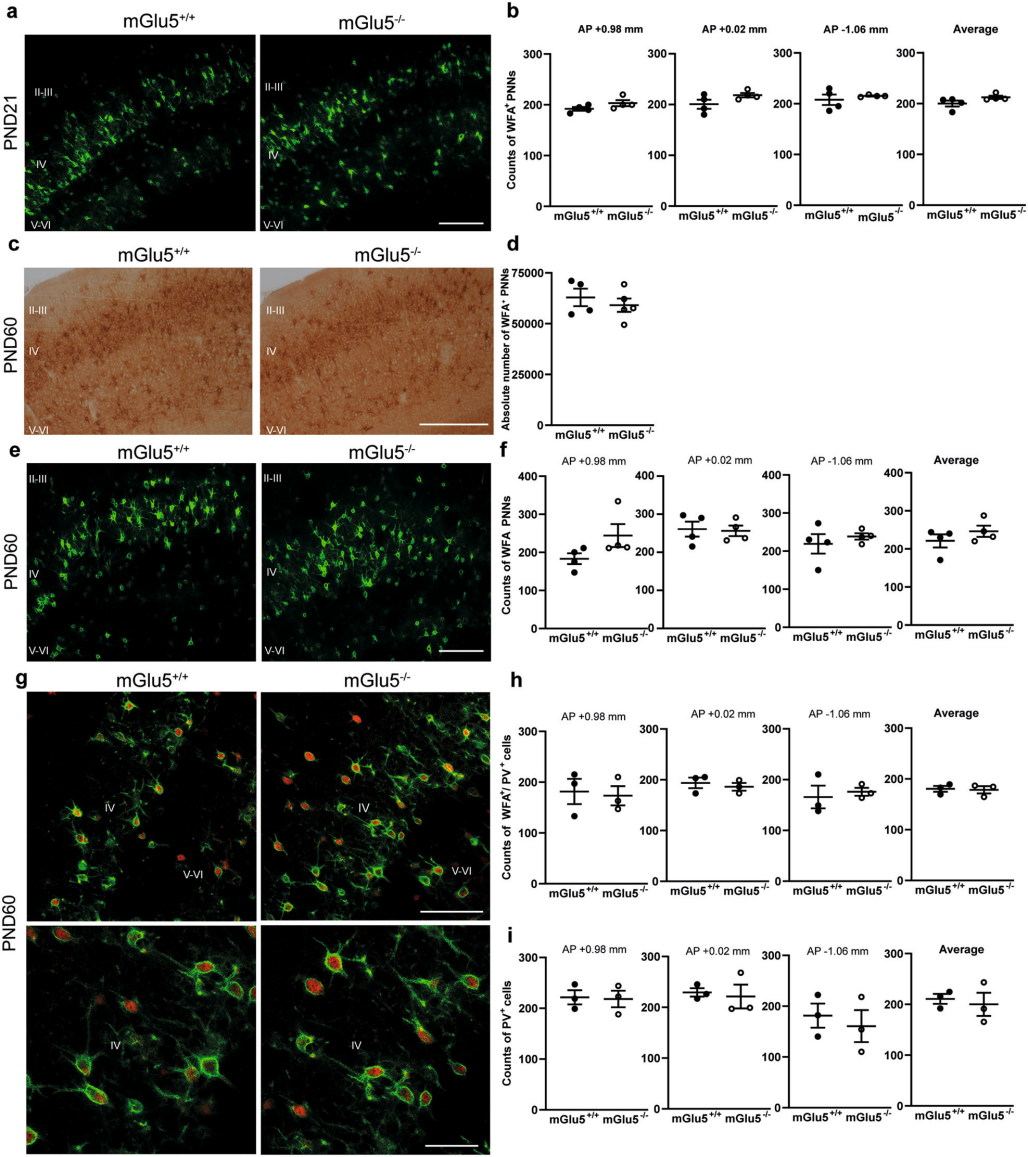
No difference in WFA^+^ PNNs between mGlu5^−/−^ and wild-type mice in the somatosensory cortex at PND21 and PND60. **a**, **b** WFA^+^ PNN density at PND21. **c**, **d** Stereological WFA^+^ PNN counting in the somatosensory cortex at PND60. **e**–**i** WFA^+^ PNN, WFA^+^/PV^+^ and PV^+^ cell density at PND60. Green = WFA; red = PV. Scale bars = 50 μm (**e** and upper panels in **g**); = 25 μm (**g** lower panels); = 100 μm (**a**, **c**). Immunostaining in **e**, **f**, in **g**, **h**, and in **i** was performed in three independent experiments.

We extended the analysis of WFA^+^ PNNs to later developmental stages. The density of WFA^+^ PNNs in the somatosensory cortex was unchanged in mGlu5^−/−^ mice at PND21 (Fig. 2a, b). At PND60, the absolute number of WFA^+^ PNNs did not differ between the two genotypes in the somatosensory cortex (Fig. 2c, d), and prefrontal cortex (Supplementary Fig. 2c, d). The lack of difference in the somatosensory cortex was confirmed by measuring the density of WFA^+^ and WFA^+^/PV^+^ cells (Fig. 2e–i). Interestingly, at PND60 a large proportion (about 86%) of WFA^+^ PNNs enwrapped PV^+^ neurons in wild-type mice (compare average counts of Fig. 2f, h), and this proportion was similar in mGlu5^−/−^ mice (Fig. 2f, h).

Thus, the developmental pattern of expression of WFA^+^ PNNs in the somatosensory cortex was anticipated in mGlu5^−/−^ mice, with levels of WFA^+^ PNNs at PND16 being close to adult levels. In contrast, expression of WFA^+^ PNNs increased linearly across development in mGlu5^+/+^ mice (Supplementary Fig. 3).

### Pharmacological blockade of mGlu5 receptors increased the density of WFA^+^ PNNs in the somatosensory cortex at PND16 but not at PND21

**Fig. 3:**
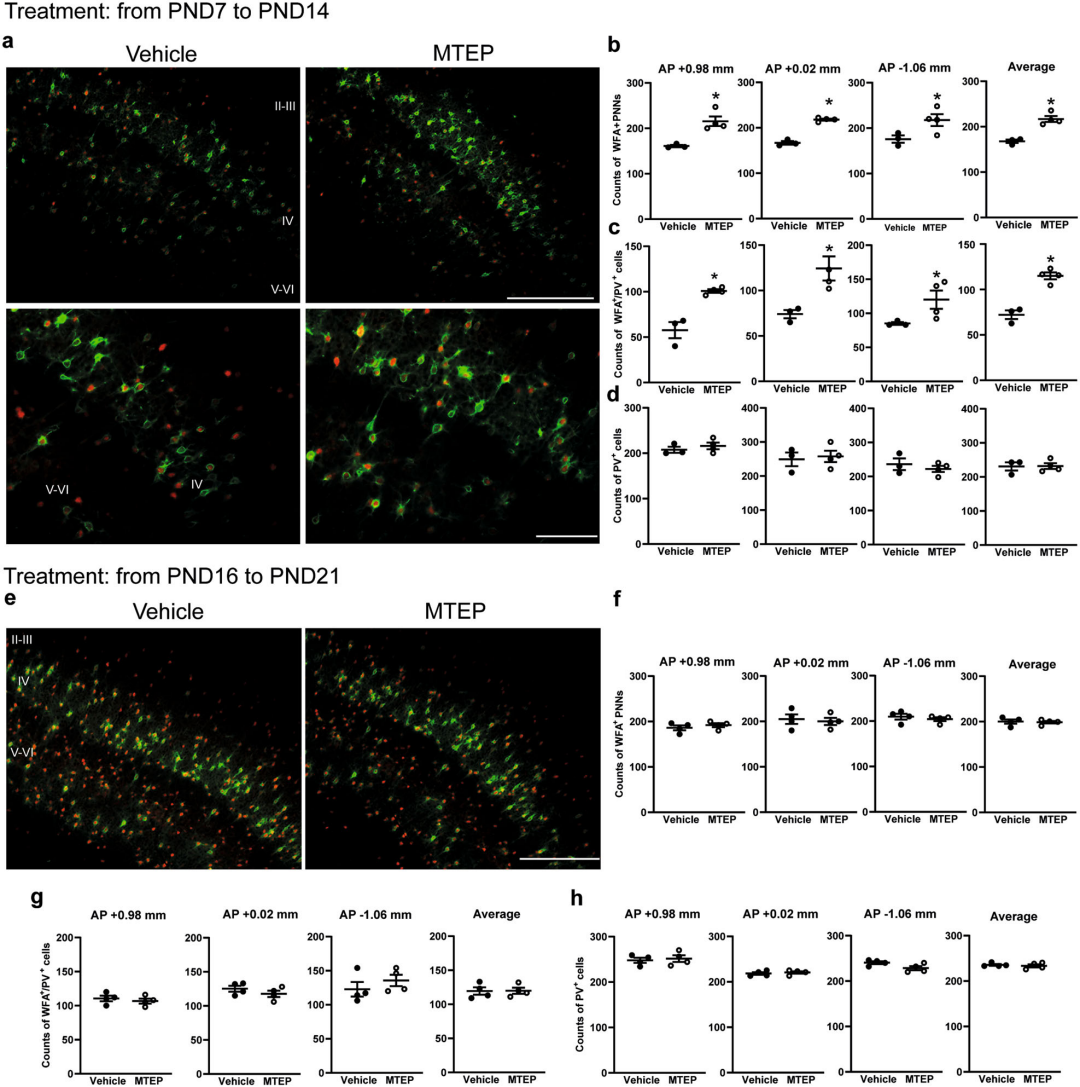
Pharmacological blockade of mGlu5 receptors increases WFA^+^ PNN density in the somatosensory cortex at PND16 but not at PND21. Mice were treated i.p. with either saline or MTEP (3 mg/kg) daily from PND7 to PND14 and killed at PND16. **a** WFA (green) and PV (red) staining in vehicle and MTEP treated mice. Scale bar = 100 μm (upper panels) and 50 μm (lower panels). **b**, **c** *Statistically significant vs. the corresponding mGlu5^+/+^ sections (Student’s *t* test); in **b** t5 = −4.248, *p* = 0.008 (AP + 0.98 mm); t5 = −12.576, *p* = <0.01 (AP + 0.02 mm); t5 = −2.473, *p* = 0.056 (AP – 1.06 mm); t5 = −5.867, *p* = 0.002 (average counts). In **c** t5 = −5.556, *p* = 0.003 (AP + 0.98 mm); t5 = −3.101, *p* = 0.027 (AP + 0.02 mm); t5 = −2.181, *p* = 0.081 (AP – 1.06 mm), t5 = −6.982, *p* < 0.001 (average counts). **e**–**h** WFA/PV immunostaining in mice treated daily with MTEP or vehicle from PND16 to PND21. **e** Scale bar = 100 μm. Immunostaining in **c**, **d** and **g**, **h** was performed in sections adjacent to the levels indicated in **b** and **f**, respectively.

To further examine the role of mGlu5 receptors in the regulation of PNNs at PND16, we treated wild-type mice with the mGlu5 receptor NAM, MTEP (3 mg/kg, i.p.), from PND7 to PND14 (mice were killed at PND16). This treatment increased the density of both WFA^+^ and WFA^+^/PV^+^ cells in the somatosensory cortex, as compared to mice treated with vehicle (Fig. 3a–d). In contrast, daily treatment with MTEP from PND16 to PND21 did not change the density of WFA^+^ and WFA^+^/PV^+^ cells in the somatosensory cortex (Fig. 3e–h), further demonstrating that the influence of mGlu5 receptors on PNN formation/degradation is restricted to a specific developmental window.

### Characterization of the mechanisms underlying the increase in PNN density in mGlu5^−/−^ mice at PND16

**Fig. 4:**
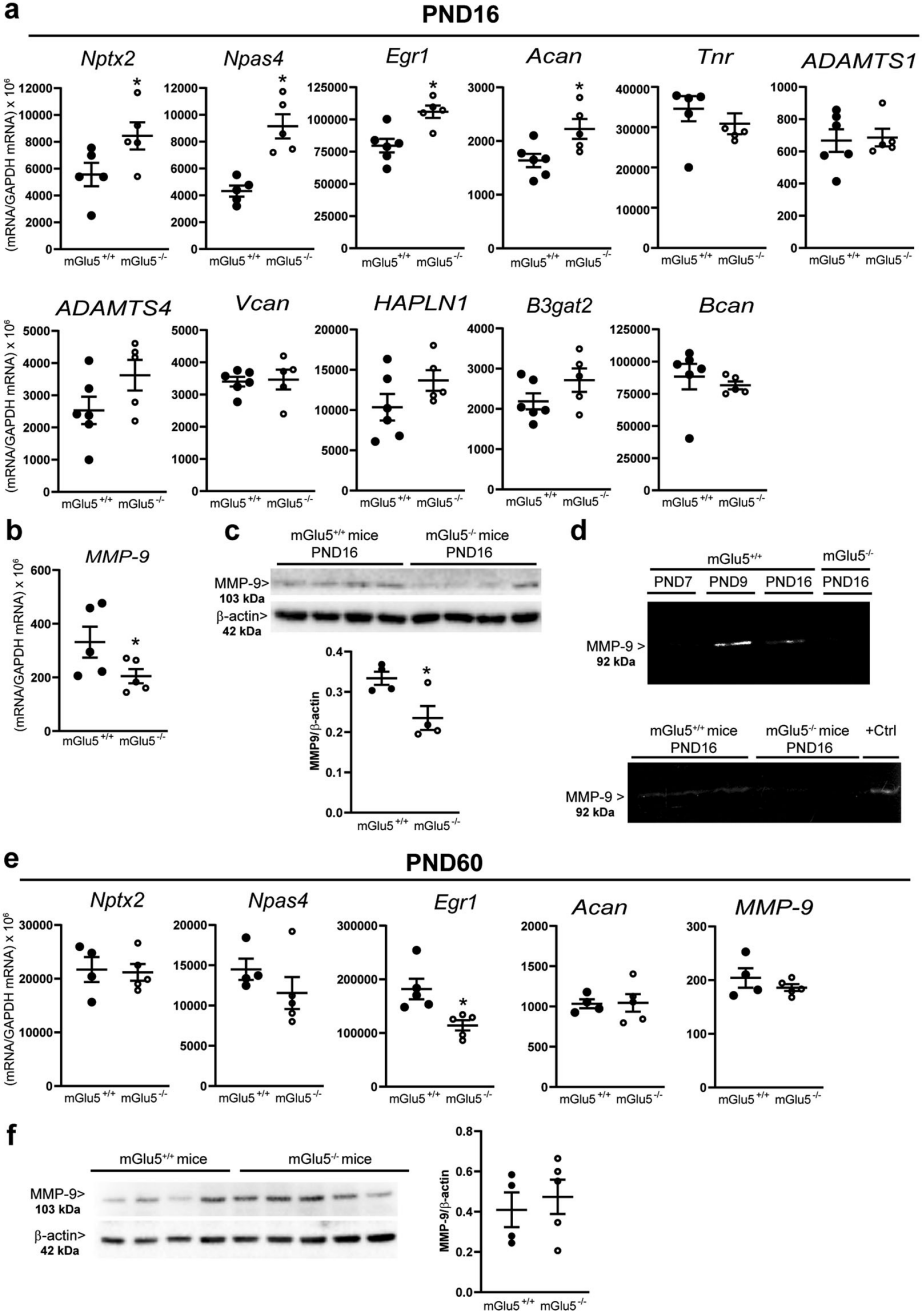
Expression of genes and proteins involved in the formation or degradation of PNNs in the somatosensory cortex of mGlu5^+/+^ and mGlu5^−/−^ mice at PND16 and PND60. **a** mRNA levels (copy numbers normalized by GAPDH) *Statistically significant vs. mGlu5^+/+^ (Student’s *t* test); Nptx2, t8 = −2.15; *p* = 0/03; NPAS4, t8 = −4.87; *p* = 0.0012; Egr1, t9 = −3.63; *p* = 0.005; Acan, t9 = −2.73; *p* = 0.02. **b**, **c** MMP-9 mRNA and protein levels. *Statistically significant vs. mGlu5^+/+^ (Student’s *t* test); **b** t8 = −2.08; *p* = 0.03; **c** t6 = 2.905; *p* = 0.027. **d** Gelatin zymography of MMP-9 activity. **e** mRNA levels of Nptx2, NPAS4, Egr-1, Acan, and MMP-9 in the somatosensory cortex of the two genotypes at PND60. *Statistically significant vs. mGlu5^+/+^ (Student’s *t* test); t8 = 3,162; *p* < 0.05; **f** Immunoblot of MMP-9 in the somatosensory cortex at PND60.

To unravel the mechanism(s) by which mGlu5 receptors control the appearance of PNNs in the developing somatosensory cortex, we examined the expression of genes involved in PNN formation or degradation at PND16. Expression of *Nptx2* (encoding the neuronal activity-regulated pentraxin or Narp), *Npas4*, *Acan* (encoding aggrecan), and *Egr1*, was increased in the somatosensory cortex mGlu5^−/−^ mice, whereas the transcripts encoding tenascin (*Tnr*), ADAMTS1 and -4, versican (*Vcan)*, brevican (*Bcan)*, link protein (*HAPLN1*) and galactosylgalctosylxylosylprotein-3-β-glucuronosyltransferase 2 (*B3gat2*) were unchanged (Fig. 4a).

Interestingly, both the transcript and protein levels of the PNN-degrading enzyme, MMP-9, were reduced in mGlu5^−/−^ mice at PND16 (Fig. 4b, c). Zymography analysis showed the expected reduction of MMP-9 activity from PND9 to PND16 in wild-type mice (Fig. 4d). MMP-9 activity was largely reduced in the somatosensory cortex of mGlu5^−/−^ mice at PND16 (Fig. 4d).

At PND60 there was no difference in the transcripts of *Nptx2*, *Npas4*, *Acan*, and MMP-9, and in MMP-9 protein levels in the somatosensory cortex of the two genotypes. The only difference was found in the transcript of *Egr-1*, which was decreased in the somatosensory cortex of mGlu5^−/−^ mice (Fig. 4e, f).

### The lack of mGlu5 receptors occludes the PNN-promoting effect of repeated sensory stimulation and unmasks a PNN-lowering effect of sensory deprivation in the developing somatosensory cortex

**Fig. 5:**
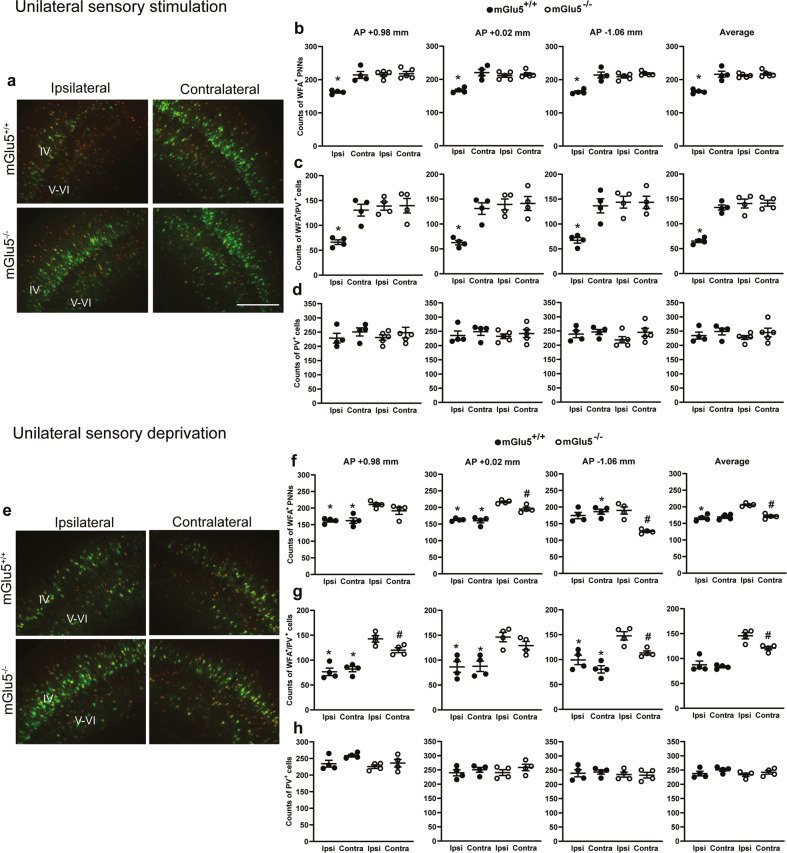
mGlu5 receptors shape the PNN response to sensory experience in the somatosensory cortex at PND16. **a**–**d** WFA^+^, WFA^+^/PV^+^, and PV^+^ cell density in the ipsilateral (Ipsi) or contralateral (Contra) somatosensory cortex of mGlu5^+/+^ and mGlu5^−/−^ mice after unilateral whisker stimulation from PND9 to PND16. **a** green = WFA and red = PV; scale bar = 100 μm. **b**, **c** *statistically significant vs the respective contralateral side. Immunostaining in **c** and **d** was performed in sections adjacent to levels indicated in **b**. **e**–**h** WFA^+^, WFA^+^/PV^+^ and PV^+^ cell density in the ipsilateral (Ipsi) or contralateral (Contra) somatosensory cortex of mGlu5^+/+^ and mGlu5^−/−^ mice after unilateral whisker trimming from PND9 to PND16. **e** green = WFA and red = PV; scale bar = 100 μm. **f**, **g** statistically significant vs the respective side in mGlu5^−/−^ mice (*) or vs the ipsilateral side of mGlu5^−/−^ mice (#). Immunostaining in **g** and **h** was performed in sections adjacent to levels indicated in **f**. Statistical analysis was performed by two-way ANOVA followed by Bonferroni post hoc test. All *F* values are in the Supplementary Materials.

We examined whether PNN expression in the somatosensory cortex could be affected by repetitive sensory stimulation (4 times per day) of the vibrissae and whether PNN plasticity under these conditions was influenced by mGlu5 receptors. We found an increased density of WFA^+^ PNNs and WFA^+^/PV^+^ cells in the contralateral somatosensory cortex in wild-type mice after unilateral sensory stimulation (Fig. 5a–c). In contrast, there was no difference between the ipsilateral and contralateral cortex of mGlu5^−/−^ mice, in which expression of PNNs in both cortices was upregulated (Fig. 5a, c). The density of PV^+^ cells did not change, as expected (Fig. 5d).

We also induced unilateral sensory deprivation by trimming all left whiskers every other day from PND9 to PND16. Following this treatment, we found no difference in PNN density between the ipsilateral and contralateral somatosensory cortex of wild-type mice. In contrast, unilateral sensory deprivation reduced the density of both WFA^+^ PNNs and WFA^+^/PV^+^ cells in the contralateral somatosensory cortex of mGlu5^−/−^ mice with respect to the ipsilateral cortex of the same genotype (reduction was significant in 2 of the 3 sections and in the average cell counts) (Fig. 5e–g). There were no changes in the density of PV^+^ cells (Fig. 5f).

## Discussion

In the first 2–3 postnatal weeks, the functional topography of the developing somatosensory cortex is shaped by activity-dependent and -independent processes that coordinate the emergence of sensory experience. After PND10-12, neuronal activity shifts from highly coordinated to sparse^36–38^ to ensure encoding of sensory information. Formation of perisomatic inhibition by PV^+^ basket cells, coincides with the opening of critical windows of cortical development^39–41^. PNNs play a key role in regulating interneuron plasticity during postnatal development, and their formation coincides with the closure of critical periods^3,29^.

It is during the first two postnatal weeks that mGlu5 receptors are highly expressed and functional in the cerebral cortex^22,23^. One of the most consistent findings in the mGlu field is the dramatic mGlu5-mediated PI hydrolysis in the cerebral cortex and other brain regions in the early postnatal life. After PND14/15, mGlu5-mediated PI hydrolysis progressively declines to become negligible in the adult life^20,23^. This characteristic, which is not shared by other G_q/11_ coupled receptors, suggests a key role for mGlu5 receptors in the regulation of brain development.

We were surprised to find that the absolute number of WFA^+^ PNNs was almost doubled in mGlu5^−/−^ mice at PND16. To our knowledge, this is one the most striking histological phenotypes ever observed in mice lacking any mGlu subtype. About 40% of WFA^+^ PNNs enwrapped PV^+^ interneurons at PND16, and the density of WFA^+^/PV^+^ cells was largely increased in mGlu5^−/−^ mice. A similar scenario was seen in mice treated with the mGlu5 NAM, MTEP, at a daily dose of 3 mg/kg from PND7 to PND14. Pharmacokinetic studies showed that MTEP has a plasma half-life of about 15 min in mice, and the drug reached a 75% of brain mGlu5 receptor occupancy at 5–15 min post-dosing, and occupancy returned back to baseline at 60 min^42^. Because treatment with MTEP increased WFA^+^ PNN density to an extent similar to that seen in mGlu5^−/−^ mice, it is possible that either drug metabolism or brain accumulation changes in response to daily administrations for 8 days, or that receptor blockade outlasted the half-life of the drug.

Taken together, our findings suggest that endogenous activation of mGlu5 receptors restrains PNN formation around PV^+^ neurons in the somatosensory cortex at PND16. Comparison of PNN density at PND9, PND16, PND21, and PND60, indicated that the absence of mGlu5 receptors anticipated the plateau of PNN expression in the somatosensory cortex.

mGlu5 receptors might control PNN expression *via* a number of non-redundant mechanisms that are involved in both PNN formation and degradation. Expression of the genes encoding Egr-1, Narp, NPAS4, and aggrecan was upregulated in mGlu5^−/−^ mice. The immediate early gene, *Egr-1*, encodes a transcription factor that is highly expressed in PV^+^ interneurons, and related to the critical period of cortical plasticity^43,44^. The egr-1 target *Narp* encodes a calcium-dependent lectin which regulates excitatory synaptic responses and supports spontaneous firing of PV^+^ interneurons^45,46^. Narp binds to hyaluronan and chondroitinsulphate E and enhances PNN formation in cortical neurons^47^. PNNs modulate mechanisms of synaptic scaling *via* their interaction with Narp, which, in turn, regulates AMPA receptor clustering in dendritic spines of PV^+^ interneurons^46^. PNNs are reduced in the auditory cortex of mice modeling Fragile X syndrome^48^, and the associated reduction in Narp expression might impair synaptic scaling in PV^+^ neurons with ensuing cortical hyperexcitability^49^. Interestingly, mGlu5 receptor signaling is enhanced in the cereberal cortex of Fragile X mice^50,51^, and this fits nicely with the hypothesis that mGlu5 receptors restrain PNN expression by negatively modulating the *Egr-1/Narp* axis.

NPAS4 is an activity-dependent trascription factor, which is induced by sensory stimuli and contributes to maturation and topographic organization of synaptic inhibition^52,53^. The enhancing effect of adolescent stress on PNN formation in the prefrontal cortex requires a full expression of NPAS4^54^. The increase in NPAS4 transcript levels found in mGlu5^−/−^ mice raises the possibility that mGlu5 receptors control the spatial organization of synaptic inhibition by restraining the expression of NPAS4 at critical temporal windows.

Aggrecan is a major structural component of PNNs and its expression is delayed after sensory deprivation^32,55,56^. Interestingly, the genes encoding for other structural PNN components physically linked to aggrecan, such as tenascin-R, versican and brevican^57^ were unaffected by the lack of mGlu5 receptors. Of note, WFA staining is absent in cortical tissue from mice lacking aggrecan^58^, whereas mice lacking versican or brevican have normal PNNs^59^. In addition, the absence of aggrecan does not affect tenascin-R, brevican, and HAPLN-1 expression^58^. This might explain why the increase in *Acan* gene expression was associated with a greater number of WFA^+^ PNNs with no changes in the expression of genes encoding other structural components. No changes were also found in the gene encoding B3GAT2, which is involved in the biosynthesis of the human natural killer-1 carbohydrate epitope of tenascin-R^60^.

The lack of mGlu5 receptors largely reduced the expression and activity of MMP-9, whereas the transcripts encoding for other PNN-degrading enzymes (ADAMTS1 and -4)^61^ were unchanged. Expression of MMP-9 in the mouse cerebral cortex peaks in the early postnatal life and declines afterward^62,63^. This ensures a low background MMP-9 activity after the first postnatal week, which is essential for the regulation of cortical plasticity. MMP-9 activity increases in response to synaptic activation^64,65^, and MMP-9 expression can by regulated by a number of signaling molecules and environmental factors during development and in the adult life^66^. Interestingly, pharmacological activation of mGlu5 receptors was shown to increase MMP-9 protein synthesis by dissociating MMP-9 mRNA from Fragile-X mental retardation protein (FMRP) in cultured neurons^65^. The lack of this mechanism may account for the decreased MMP-9 expression found in the somatosensory cortex of mGlu5^−/−^ mice. Our data offer a direct demonstration that mGlu5 receptors support MMP-9 expression and activity in the developing somatosensory cortex. We wish to highlight that changes in the expression of genes or proteins involved in PNN formation and degradation are only correlative and do not demonstrate a mechanism by which mGlu5 receptors regulate PNN density at PND16. However, the lack of changes in the transcripts encoding Narp, NPAS4, Acan, and MMP-9 at PND60 (when PNN density was unchanged in mGlu5^−/−^ mice) suggests that these factors might be involved in the regulation of PNNs by mGlu5 receptors.

The evidence that mGlu5 receptors are involved in the formation of somatosensory map in the barrel cortex^34,67^ gave us the impetus to investigate whether changes in PNN expression caused by sensory stimulation were under the control of mGlu5 receptors. Early sensory deprivation caused by whisker trimming was found to reduce Cat-315^+^ PNN density in the barrel cortex^32,55^. Continuous whisker trimming starting from PND0 did not change WFA^+^ PNN density at PND30 in one study^32^, but reduced WFA^+^ PNN density in layer IV of the barrel cortex at PND28 and PND56 in another study^55^. We adopted an opposite strategy by performing unilateral whisker stimulations from PND9 to PND16. We have shown for the first time that sensory stimulation enhances WFA^+^ PNN formation in the contralateral somatosensory cortex. Interestingly, this effect was occluded by the constitutive hyperexpression of WFA^+^ PNNs in mGlu5^−/−^ mice. This suggests that one of the functions of mGlu5 receptors is to lower background levels of PNN expression to ensure an optimal signal-to-noise ratio of PNN formation in response to sensory stimulation. This hypothesis was supported by data obtained in mice subjected to eight days of unilateral sensory deprivation from PND9 to PND16. In wild-type mice, this treatment did not change the density of WFA^+^ PNN in the somatosensory cortex. This was largely expected because whisker trimming in mice every other day since PND2 was shown to reduce PNN expression in layer IV of the cortex at PND28 and PND56, but not at PND14 and PND21^32^. However, a significant reduction was seen in the contralateral somatosensory cortex of mGlu5^−/−^ mice, in which the constitutive expression of WFA^+^ PNNs was higher. All together, these findings suggest that mGlu5 receptors shape the PNN response to sensory experience during postnatal development.

In conclusion, our findings disclose a novel mechanism of PNN regulation in the somatosensory cortex, and suggest that mGlu5 receptors play a key role in developmental plasticity of PV^+^ interneurons. mGlu5 receptors are candidate drug targets for the treatment of schizophrenia^68,69^ and autism spectrum disorders^70–72^, two neurodevelopmental disorders characterized by a dysfunction of GABAergic interneurons^73,74^. Our data may lay the groundwork for the study of the mGlu5/PNN axis in these disorders.

## Supplementary information

Supplementary Materials

Supplementary Figure 1

Supplementary Figure 2

Supplementary Figure 3

## References

[1] Testa D, Prochiantz A, Di Nardo AA (2019). Perineuronal nets in brain physiology and disease. Semin Cell Dev. Biol..

[2] Zaremba S, Guimaraes A, Kalb RG, Hockfield S (1989). Characterization of an activity-dependent, neuronal surface proteoglycan identified with monoclonal antibody Cat-301. Neuron.

[3] Pizzorusso T (2002). Reactivation of ocular dominance plasticity in the adult visual cortex. Science.

[4] Takesian AE, Hensch TK (2013). Balancing plasticity/stability across brain development. Prog. Brain Res..

[5] Miyata S, Komatsu Y, Yoshimura Y, Taya C, Kitagawa H (2012). Persistent cortical plasticity by upregulation of chondroitin 6-sulfation. Nat. Neurosci..

[6] Hou X (2017). Chondroitin sulfate is required for onset and offset of critical period plasticity in visual cortex. Sci. Rep..

[7] Pantazopoulos H, Woo TU, Lim MP, Lange N, Berretta S (2010). Extracellular matrix-glial abnormalities in the amygdala and entorhinal cortex of subjects diagnosed with schizophrenia. Arch. Gen. Psychiatry.

[8] Berretta S (2012). Extracellular matrix abnormalities in schizophrenia. Neuropharmacology.

[9] Berretta S, Pantazopoulos H, Markota M, Brown C, Batzianouli ET (2015). Losing the sugar coating: potential impact of perineuronal net abnormalities on interneurons in schizophrenia. Schizophr. Res.

[10] Enwright JF (2016). Reduced labeling of parvalbumin neurons and perineuronal nets in the dorsolateral prefrontal cortex of subjects with schizophrenia. Neuropsychopharmacology.

[11] Mercuri FA, Maciewicz RA, Tart J, Last K, Fosang AJ (2000). Mutations in the interglobular domain of aggrecan alter matrix metalloproteinase and aggrecanase cleavage patterns. Evidence that matrix metalloproteinase cleavage interferes with aggrecanase activity. J. Biol. Chem..

[12] Madsen SH (2010). Aggrecanase- and matrix metalloproteinase-mediated aggrecan degradation is associated with different molecular characteristics of aggrecan and separated in time ex vivo. Biomarkers.

[13] Lovelace JW (2016). Matrix metalloproteinase-9 deletion rescues auditory evoked potential habituation deficit in a mouse model of Fragile X Syndrome. Neurobiol. Dis..

[14] Wen TH (2018). Genetic reduction of matrix metalloproteinase-9 promotes formation of perineuronal nets around parvalbumin-expressing interneurons and normalizes auditory cortex responses in developing Fmr1 knock-out mice. Cereb. Cortex.

[15] Kwok JC, Carulli D, Fawcett JW (2010). In vitro modeling of perineuronal nets: hyaluronan synthase and link protein are necessary for their formation and integrity. J. Neurochem..

[16] Nicoletti F (2011). Metabotropic glutamate receptors: from the workbench to the bedside. Neuropharmacology.

[17] Reimers S, Hartlage-Rübsamen M, Brückner G, Rossner S (2007). Formation of perineuronal nets in organotypic mouse brain slice cultures is independent of neuronal glutamatergic activity. Eur. J. Neurosci..

[18] Cardis R, Cabungcal JH, Dwir D, Do KQ, Steullet P (2018). A lack of GluN2A-containing NMDA receptors confers a vulnerability to redox dysregulation: Consequences on parvalbumin interneurons, and their perineuronal nets. Neurobiol. Dis..

[19] Imbriglio T (2019). Developmental abnormalities in cortical GABAergic system in mice lacking mGlu3 metabotropic glutamate receptors. FASEB J..

[20] Nicoletti F, Iadarola MJ, Wroblewski JT, Costa E (1986). Excitatory amino acid recognition sites coupled with inositol phospholipid metabolism: developmental changes and interaction with alpha1-adrenoceptors. Proc. Natl Acad. Sci. USA.

[21] Romano C, Van Den Pol AN, O’Malley KL (1996). Enhanced early developmental expression of the metabotropic glutamate receptor mGluR5 in rat brain: protein, mRNA splice variants, and regional distribution. J. Comp. Neurol..

[22] Catania MV, Aronica E, Sortino MA, Canonico PL, Nicoletti F (1991). Desensitization of metabotropic glutamate receptors in neuronal cultures. J. Neurochem.

[23] Di Menna L (2018). Functional partnership between mGlu3 and mGlu5 metabotropic glutamate receptors in the central nervous system. Neuropharmacology.

[24] Barnes SA (2015). Convergence of hippocampal pathophysiology in syngap+/- and Fmr1-/y mice. J. Neurosci..

[25] Luoni A (2018). Altered expression of schizophrenia-related genes in mice lacking mGlu5 receptors. Eur. Arch. Psychiatry Clin. Neurosci..

[26] Doherty AJ, Palmer MJ, Henley JM, Collingridge GL, Jane DE (1997). (RS)-2-chloro-5-hydroxyphenylglycine (CHPG) activates mGlu5, but no mGlu1, receptors expressed in CHO cells and potentiates NMDA responses in the hippocampus. Neuropharmacology.

[27] Awad H, Hubert GW, Smith Y, Levey AI, Conn PJ (2000). Activation of metabotropic glutamate receptor 5 has direct excitatory effects and potentiates NMDA receptor currents in neurons of the subthalamic nucleus. J. Neurosci..

[28] Moghaddam B, Javitt D (2012). From revolution to evolution: the glutamate hypothesis of schizophrenia and its implication for treatment. Neuropsychopharmacology.

[29] Nowicka D, Liguz-Lecznar M, Skangiel-Kramska J (2003). A surface antigen delineating a subset of neurons in the primary somatosensory cortex of the mouse. Acta Neurobiol. Exp..

[30] Nakamura M (2009). Expression of chondroitin sulfate proteoglycans in barrel field of mouse and rat somatosensory cortex. Brain Res..

[31] Karetko-Sysa M, Skangiel-Kramska J, Nowicka D (2014). Aging somatosensory cortex displays increased density of WFA-binding perineuronal nets associated with GAD-negative neurons. Neuroscience.

[32] Ueno H, Suemitsu S, Okamoto M, Matsumoto Y, Ishihara T (2017). Sensory experience-dependent formation of perineuronal nets and expression of Cat-315 immunoreactive components in the mouse somatosensory cortex. Neuroscience.

[33] Chu P (2018). The impact of perineuronal net digestion using chondroitinase ABC on the intrinsic physiology of cortical neurons. Neuroscience.

[34] Ballester-Rosado CJ, Sun H, Huang JY, Lu HC (2016). mGluR5 exerts cell-autonomous influences on the functional and anatomical development of layer iv cortical neurons in the mouse primary somatosensory cortex. J. Neurosci..

[35] Horii-Hayashi N, Sasagawa T, Matsunaga W, Nishi M (2015). Development and structural variety of the chondroitin sulfate proteoglycans-contained extracellular matrix in the mouse brain. Neural Plast..

[36] Golshani P. et al. Internally mediated developmental desynchronization of neocortical network activity. *J. Neurosci.* **29**, 10890-10899 (2009). 10.1523/JNEUROSCI.2012-09.2009 PMC2771734 19726647

[37] Rochefort NL (2009). Sparsification of neuronal activity in the visual cortex at eye-opening. Proc. Natl Acad. Sci. USA.

[38] Wolfe J, Houweling AR, Brecht M (2010). Sparse and powerful cortical spikes. Curr. Opin. Neurobiol..

[39] De Marco García NV, Karayannis T, Fishell G (2011). Neuronal activity is required for the development of specific cortical interneuron subtypes. Nature.

[40] Wamsley B, Fishell G (2017). Genetic and activity-dependent mechanisms underlying interneuron diversity. Nat. Rev. Neurosci..

[41] Toyoizumi T (2013). A theory of the transition to critical period plasticity: inhibition selectively suppresses spontaneous activity. Neuron.

[42] Anderson JJ (2003). In vivo receptor occupancy of mGlu5 receptor antagonists using the novel radioligand [3H]3-methoxy-5-(pyridin-2-ylethynyl)pyridine. Eur. J. Pharmacol..

[43] Mataga N, Fujishima S, Condie BG, Hensch TK (2001). Experience-dependent plasticity of mouse visual cortex in the absence of the neuronal activity-dependent marker egr1/zif268. J. Neurosci..

[44] Mower GD (2002). Kaplan IV. Immediate early gene expression in the visual cortex of normal and dark reared cats: differences between fos and egr-1. Brain Res Mol. Brain Res.

[45] O’Brien RJ1 (1999). Synaptic clustering of AMPA receptors by the extracellular immediate-early gene product Narp. Neuron.

[46] Chang MC (2010). Narp regulates homeostatic scaling of excitatory synapses on parvalbumin-expressing interneurons. Nat. Neurosci..

[47] Van’t Spijker HM (2019). Neuronal pentraxin 2 binds PNNs and enhances PNN formation. Neural Plast..

[48] Lovelace JW (2020). Deletion of Fmr1 from forebrain excitatory neurons triggers abnormal cellular, EEG, and behavioral phenotypes in the auditory cortex of a mouse model of fragile X syndrome. Cereb. Cortex.

[49] Wen TH, Binder DK, Ethell IM, Razak KA (2018). The perineuronal ‘safety’ net? Perineuronal net abnormalities in neurological disorders. Front Mol. Neurosci..

[50] Hays SA, Huber KM, Gibson JR (2011). Altered neocortical rhythmic activity states in Fmr1 KO mice are due to enhanced mGluR5 signaling and involve changes in excitatory circuitry. J. Neurosci..

[51] Guo W (2016). Selective disruption of metabotropic glutamate receptor 5-homer interactions mimics phenotypes of fragile X syndrome in mice. J. Neurosci..

[52] Lin Y (2008). Activity-dependent regulation of inhibitory synapse development by Npas4. Nature.

[53] Bloodgood BL, Sharma N, Browne HA, Trepman AZ, Greenberg ME (2013). The activity-dependent transcription factor NPAS4 regulates domain-specific inhibition. Nature.

[54] Page CE, Alexander J, Shepard R, Coutellier L (2018). Npas4 deficiency interacts with adolescent stress to disrupt prefrontal GABAergic maturation and adult cognitive flexibility. Genes Brain Behav..

[55] McRae PA, Rocco MM, Kelly G, Brumberg JC, Matthews RT (2007). Sensory deprivation alters aggrecan and perineuronal net expression in the mouse barrel cortex. J. Neurosci..

[56] Ye Q, Miao QL (2013). Experience-dependent development of perineuronal nets and chondroitin sulfate proteoglycan receptors in mouse visual cortex. Matrix Biol..

[57] Sorg BA (2016). Casting a wide net: role of perineuronal nets in neural plasticity. J. Neurosci..

[58] Giamanco KA, Morawski M, Matthews RT (2010). Perineuronal net formation and structure in aggrecan knockout mice. Neuroscience.

[59] Dours-Zimmermann MT (2009). Versican V2 assembles the extracellular matrix surrounding the nodes of ranvier in the CNS. J. Neurosci..

[60] Kähler AK1 (2011). Candidate gene analysis of the human natural killer-1 carbohydrate pathway and perineuronal nets in schizophrenia: B3GAT2 is associated with disease risk and cortical surface area. Biol. Psychiatry.

[61] Bozzelli PL, Alaiyed S, Kim E, Villapol S, Conant K (2018). Proteolytic remodeling of perineuronal nets: effects on synaptic plasticity and neuronal population dynamics. Neural Plast..

[62] Bednarek N (2009). Ontogeny of MMPs and TIMPs in the murine neocortex. Pediatr. Res..

[63] Reinhard SM, Razak K, Ethell IM (2015). A delicate balance: role of MMP-9 in brain development and pathophysiology of neurodevelopmental disorders. Front Cell Neurosci..

[64] Gawlak M (2009). High resolution in situ zymography reveals matrix metalloproteinase activity at glutamatergic synapses. Neuroscience.

[65] Janusz A (2013). The fragile X mental retardation protein regulates matrix metalloproteinase 9 mRNA at synapses. J. Neurosci..

[66] Van den Steen PE (2002). Biochemistry and molecular biology of gelatinase B or matrix metalloproteinase-9 (MMP-9). Crit. Rev. Biochem Mol. Biol..

[67] Ballester-Rosado CJ (2010). mGluR5 in cortical excitatory neurons exerts both cell-autonomous and -nonautonomous influences on cortical somatosensory circuit formation. J. Neurosci..

[68] Bruno V (2017). The impact of metabotropic glutamate receptors into active neurodegenerative processes: A “dark side” in the development of new symptomatic treatments for neurologic and psychiatric disorders. Neuropharmacology.

[69] Stansley BJ, Conn PJ (2018). The therapeutic potential of metabotropic glutamate receptor modulation for schizophrenia. Pharm. Curr. Opin..

[70] Lüscher C, Huber KM (2010). Group 1 mGluR-dependent synaptic long-term depression: mechanisms and implications for circuitry and disease. Neuron.

[71] Krueger DD, Bear MF (2011). Toward fulfilling the promise of molecular medicine in fragile X syndrome. Annu Rev. Med.

[72] D’Antoni S (2014). Dysregulation of group-I metabotropic glutamate (mGlu) receptor mediated signalling in disorders associated with intellectual disability and autism. Neurosci. Biobehav Rev..

[73] Hashimoto T (2008). Alterations in GABA-related transcriptome in the dorsolateral prefrontal cortex of subjects with schizophrenia. Mol. Psychiatry.

[74] Glausier JR, Fish KN, Lewis DA (2014). Altered parvalbumin basket cell inputs in the dorsolateral prefrontal cortex of schizophrenia subjects. Mol. Psychiatry.

