# Supplementary material for: Perineuronal nets are under the control of type-5 metabotropic glutamate receptors in the developing somatosensory cortex
Source: Transl Psychiatry. 2021 Feb 18;11:109. doi: 10.1038/s41398-021-01210-3 (PMC7889908; doi:10.1038/s41398-021-01210-3)
Supplement: Supplementary file 1 — Supplementary Materials [file 41398_2021_1210_MOESM1_ESM.docx]

***Supplementary materials***

*Immunofluorescent staining*

Mice were anesthesized and subjected to transcardial perfusion with saline followed by ice-cold 4% paraformaldehyde in PBS, pH 7.4. Brains were removed, post-fixed overnight in the same fixative, and then placed in 30% sucrose 0.1 M PBS for 24 h. Free-floating brain slices (30 μm) were rinsed in PBS, quenched in 3% H_2_O_2_ and 10% methanol for 15 min, and blocked in 5% normal donkey serum and 0.3% Triton X-100 in PBS for 2 hours at room temperature. For PNN detection in the somatosensory cortex, we stained sections with *Wisteria Floribunda Agglutinin* (WFA), a lectin that binds selectively N-acetylgalactosamines-β1 residues of glycoproteins within the extracellular matrix. WFA fluorescent staining was performed using biotin-conjugate WFA (1:1000; #L1516, Sigma-Aldrich, Germany) and fluorescent secondary antibodies (Streptavidin Alexa Fluor 488, 1:200, Invitrogen, Carsband, CA). We also performed double immunofluorescent staining for WFA and PV (monoclonal rabbit anti-PV antibodies; 1:1000, Swant, Switzerland; #PV27) using secondary Alexa Fluor 488 antibodies (1:200) and donkey anti rabbit Cy3 secondary antibodies (1:200, Jackson ImmunoResearch, Cambridge, UK). Sections were examined with a ZEISS 780 confocal laser scanning microscope, a Zeiss Carl Apotome2 microscopy (Zeiss, Gottingen, Germany) and a Thunder Imaging System by Leica Microsystem ([Wetzlar, Germany](https://www.google.com/search?sxsrf=ALeKk00X-rES966LC1NAVnKMcRRixILTmw:1587737786002&q=Wetzlar&stick=H4sIAAAAAAAAAOPgE-LUz9U3MDQvSclVgjAts4yrtLSyk63084vSE_MyqxJLMvPzUDhWGamJKYWliUUlqUXFi1jZw1NLqnISi3awMgIAc5QoIlEAAAA&sa=X&ved=2ahUKEwj7nuyVoIHpAhVE3aQKHSaPBJ4QmxMoATAUegQIBRAD&sxsrf=ALeKk00X-rES966LC1NAVnKMcRRixILTmw:1587737786002)) and processed with ZEN software and/or LAS X software. Cell counting was performed unilaterally in three coronal sections (+0.98, +0.02, and -1.06 mm from Bregma), and counted at 10x magnification.

*Stereological counting of WFA^+^ PNNs in the somatosensory cortex and prefrontal cortex*

Mice were killed by decapitation, and brains were fixed in Carnoy’s solution (ethanol 60%, acetic acid 10%, and chloroform 30%), and included in paraffin. Tissue sections (20 μm) were incubated overnight with biotin-conjugated WFA (1:500, #L1516, Sigma-Aldrich). For detection, 3,3-diaminobenzidine tetrachloride (Millipore-Sigma) was used. The number of WFA^+^ PNNs in the whole extension of the somatosensory cortex and medial prefrontal cortex (containing cingulate cortex area 1, prelimbic and infralimbic cortex) was assessed by stereological technique and optical fractionator using a Zeiss Axio Imager M1 microscope equipped with a motorized stage, a focus control system (Zeta axis), and a digital video camera. The software Image-Pro Plus 6.2 for Windows (Media Cybernetics, Rockville, MD, USA) equipped with a Macro was used for the analysis of digital images. The Macro was obtained by Imagine and Computer (Milan, Italy). For the somatosensory cortex of PND16 mice the analysis was performed on 8 sections of 20 μm, sampled every 180 μm in the rostrocaudal extension of the somatosensory cortex using a grid of disectors with a counting frame of 50 × 50 μm, and a grid size (distance between disectors center to center) of 300 × 300 μm. For the somatosensory cortex of PND60 mice the analysis was performed on 9 sections of 20 μm sampled every 180 μm. For the medial prefrontal cortex of PND16 and PND60 mice the analysis was performed on 3 sections of 20 μm, sampled every 200 μm in the rostrocaudal extension of the medial prefrontal cortex using a grid of disectors with a counting frame of 40 × 40 μm, and a grid size (distance between disectors center to center) of 200 × 200 μm. The total number of WFA^+^ PNNs was computed according to the formula: N = Σ(n) × 1/SSF × 1/ASF × 1/TSF, where n is the total number of cells counted on each disector; SSF (fraction of sections sampled) is the number of regularly spaced sections used for counts divided by the total number of sections across the areas; ASF (area sampling frequency) is the disector area divided by the area between disectors (disector area × disector number/region area); and TSF (thickness sampling frequency) is the disector thickness divided by the section thickness. The Cavalieri estimator method was used to evaluate the volume of the somatosensory cortex.

*Immunoblot analysis*

Mice at PND16 were killed by decapitation, and the somatosensory cortex was dissected and stored frozen. Tissue was homogenized at 4°C in a buffered solution composed of Tris-HCl pH 7.5, 10 mM; NaCl, 150 mM; SDS 10 %, EDTA, 5 mM; PMSF, 10 mM; IGEPAL, 1 %; leupeptin, 1 μg/ml; and aprotinin, 1 μg/ml. Equal amounts of proteins (20 μg) from supernatants were separated by 10 % SDS polyacrylamide for MMP-9 protein and 12% for PV protein. After separation, proteins were transferred on immuno-blot PVDF membranes. Membranes were incubated with the following primary antibodies: rabbit polyclonal anti-MMP-9 (Abcam, #ab38898; 1:1000), rabbit polyclonal anti-PV (Swant, Switzerland, #PV27, 1:1000) and mouse monoclonal anti-β-actin (Sigma-Aldrich, St. Louis, MO, #A5316; 1:50.000), and then incubated for 1 hour with the corresponding secondary antibodies. Immunostaining was revealed by the enhanced ECL Western blotting analysis system (Hybond ECL, GE Healthcare Europe) and by the Chemidoc computerized densitometer (Bio-Rad), quantified by ImageLab 3.0 software (Bio-Rad).

*Zymographic analysis of MMP-9 activity*

Tissue was homogenized in extraction buffer (Tris-HCl pH 7.5, 10 mM; NaCl, 150 mM; SDS 10 %, PMSF, 10 mM; IGEPAL, 1 %; leupeptin, 1 μg/ml; and aprotinin), centrifuged at 13,000 x g, and the pellet was discarded. Proteins were separated under non denaturing conditions in 10% polyacrilamide gel containing gelatin (3 mg/ml). After electrophoresis, gels were washed for 30 min in a buffer containing Triton X-100, followed by overnight incubation in developing solution (Tris 50 mM, NaCl 200 mM, CaCl2 5 mM, BriJ 0.025, pH 7.8-8) at 37°C. Gels were stained with Coomassie Blue 0.5% for 1 hour and destained by acetic acid in methanol and H_2_O (200:40:160) for 1 hour to visualize bands with gelatinolytic activity.

*Gene expression analysis*

Mice were killed by decapitation at PND16. The brains were removed and the somatosensory cortex was dissected and frozen on liquid nitrogen. Total RNA was extracted using the trizol reagent (Invitrogen, Carlsbad, CA) according to manufacturer’s instructions. The RNA was further treated with DNAse (Qiagen, Hilden, Germany), and single strand cDNA was synthesized from 1.5 μg of total RNA using Superscript III (Invitrogen) and random hexamers. Real-time PCR was performed on 15 ng of cDNA by using specific primers and Power SYBR Green Master Mix (Biorad, Hercules, CA) on an Applied Biosystems Step-One instrument. Thermal cycler conditions were as follows: 10 min at 95°C, 40 cycles of denaturation (15 sec at 95°C), and combined annealing/extension (1 min at 58-60°C). mRNA copy number for each gene was calculated from serially diluted standard curves simultaneously amplified with the samples and normalized with respect to the Glyceraldehyde-3-Phosphate Dehydrogenase (GAPDH) mRNA copy number using the following formula: (X mRNA/GAPDH mRNA) x 10^6^. Each sample was analyzed in duplicate together with two negative controls. We used the following primers Nptx2 Forw 5’AGCTCCGCACAAATGTGTCT 3’ Rev 5’GAATGCACTGTTGCCTCGCT 3’ ; Npas4 Forw 5’ ACACTCGCAAGGGTGTCTTC 3’ Rev 5’GCAACCAGGTCCACCATAGA 3’; Egr1 Forw 5’ GACCACAGAGTCCTTTTCTGACA 3’ Rev 5’ TGAAAAGGGGTTCAGGCCAC 3’; MMP9 Forw 5’CCAGCCGACTTTTGTGGTCT 3’ Rev 5’ TGGCCTTTAGTGTCTGGCTG 3’; Bcan Forw 5’TCAAAGGGGTCGTCTTCCTC 3’ Rev 5’ATCCATGTCTCCAGAGCAGG 3’; Vcan Forw 5’GGATCATCTGGATGGCGATGT 3’ Rev 5’AGCCGTAATCGCATTGGTCA 3’; Tnr Forw 5’ CCTACCAGCTGCTGTTTCGT 3’ Rev 5’ AGTTGATGCAGACACCCAGG 3’; ADAMTS1 Forw 5’ TGGACACGGGGAATGTTTGAT 3’ Rev 5’ AGTACATGTGCTGGCTGCAT 3’; B3GAT2 Forw 5’ CTGGTGAGCAGCTTCCTAGC 3’ Rev 5’ CGAGTGGTCCTCATCTCCTG 3’; HAPLN1 Forw 5’TCCCTGGTCATCACGGATCT 3’Rev 5’ATTGTAGCGTCCCAGTCGTG3’; Acan Forw 5’GATACTCTCTGACATTTGAGG3’ Rev 5’GTATCTGACGGTCTGGTCCT 3’ ADAMTS4 Forw 5’ AACCAAGCGCTTCGCTTCTCT 3’ Rev 5’GCAGGTAGCGCTTTAACCCT 3’ and GAPDH Forw 5’ CGTCCCGTAGACAAAATGGT 3’ Rev 5’ TCAATGAAGGGGTCGTTGAT 3’.

*F values of two-way ANOVA analysis + Bonferroni’s t test in Fig. 5*

In **b**: AP +0.98 mm, cortical side: F_1,14_ = 16.53, p = 0.001; genotype: F_1,14_ = 18.477, p<0.001; side x genotype: F_1,14_ = 13.729; p = 0.002; AP +0.02 mm, cortical side: F_1,14_ = 24.238, p = <0.001; genotype: F_1,14_ = 11.161, p = 0.005; side x genotype: F_1,14_ = 17.746; p<0.001; AP - 1.06 mm, cortical side: F_1,14_ = 35.74, p<0.001; genotype: F_1,14_ = 25.074, p<0.001; side x genotype: F_1,14_ = 17.406; p<0.001; average, cortical side: F_1,14_ = 31.266, p<0.001; genotype: F_1,14_ = 23.081, p<0.001; side x genotype: F_1,14_ = 21.069; p<0.001. In **c:** AP + 0.98 mm, cortical side: F_1,12_ = 9.527, p = 0.009; genotype: F_1,12_ = 14.978, p = 0.002; side x genotype: F_1,12_ = 9.093; p = 0.011; AP + 0.02 mm, cortical side: F_1,12_ = 10.774, p = 0.007; genotype: F_1,12_ = 16.198, p<0.002; side x genotype: F_1,12_ = 9.59; p = 0.009; AP – 1.06 mm, cortical side: F_1,12_ = 9.089, p = 0.011; genotype: F_1,12_ = 13.311, p<0.003; side x genotype: F_1,12_ = 9.222; p = 0.001; average, cortical side: F_1,12_ = 30.526, p<0.001; genotype: F_1,12_ = 46.376, p<0.001; side x genotype: F_1,12_ = 29.110; p<0.001.

In **f**: AP +0.98 mm, cortical side: F_1,12_ = 1.607, p = 0.229; genotype: F_1,12_ = 27.994, p<0.001; side x genotype: F_1,12_ = 1.694; p = 0.217; AP +0.02 mm, cortical side: F_1,12_ = 7.872, p = 0.016; genotype: F_1,12_ = 105.052, p<0.001; side x genotype: F_1,12_ = 4.489; p = 0.056; AP - 1.06 mm, cortical side: F_1,12_ = 9.790, p = 0.009; genotype: F_1,12_ = 7.158, p = 0.020; side x genotype: F_1,12_ = 20.425; p<0.001; average, cortical side: F_1,12_ = 17.944, p = 0.001; genotype: F_1,12_ = 30.332, p<0.001; side x genotype: F_1,12_ = 25.190; p<0.001. In **g:** AP + 0.98 mm, cortical side: F_1,12_ = 1.997, p = 0.183; genotype: F_1,12_ = 72.950, p<0.001; side x genotype: F_1,12_ = 5.357; p = 0.039; AP + 0.02 mm, cortical side: F_1,12_ = 0.658, p = 0.433; genotype: F_1,12_ = 26.492, p<0.001; side x genotype: F_1,12_ = 0.880; p = 0.367; AP – 1.06 mm, cortical side: F_1,12_ = 12.252, p = 0.004; genotype: F_1,12_ = 28.524, p<0.001; side x genotype: F_1,12_ = 1.005; p = 0.336; average, cortical side: F_1,12_ = 7.654, p = 0.017; genotype: F_1,12_ = 84.226, p<0.001; side x genotype: F_1,12_ = 3.922; p = 0.071.

**Legends of Supplementary Figures**

**Fig. S1 –** Expression of WFA^+^ PNNs in the somatosensory cortex of mGlu5^+/+^ and mGlu5^-/-^ mice at PND9.

1. WFA in green. Scale bar = 50 μm.

**Fig. S2** – Stereological counting of WFA^+^ PNNs in the medial prefrontal cortex of mGlu5^+/+^ and mGlu5^-/-^ mice at PND16 (**a,b**) and PND60 (**c,d**).

Cell counting was performed unilaterally in the region highlighted in grey containing area 1 of the cingulate cortex, and the infralimbic and prelimbic cortex.

**Fig. S3 –** Developmental pattern of expression of WFA^+^ PNNs in the somatosensory cortex of mGlu5^+/+^ and mGlu5^-/-^ mice.

The blot was constructed using the average density data of WFA^+^  cells in the four developmental timepoints. Values are means + S.E.M. of 4 determinations per group. Two way ANOVA + Bonferroni *post hoc* test. Genotype: F_1,24_ = 13.299, p = 0.001; time: F_3,24_ = 125.858, p<0.001; time x genotype: F_3,24_ = 2.912; p = 0.055; *p<0.001 *vs* mGlu5^+/+^ mice at PND16.
