# Supplementary figures and images for: Perineuronal nets are under the control of type-5 metabotropic glutamate receptors in the developing somatosensory cortex
Source: Transl Psychiatry. 2021 Feb 18;11:109. doi: 10.1038/s41398-021-01210-3 (PMC7889908; doi:10.1038/s41398-021-01210-3)

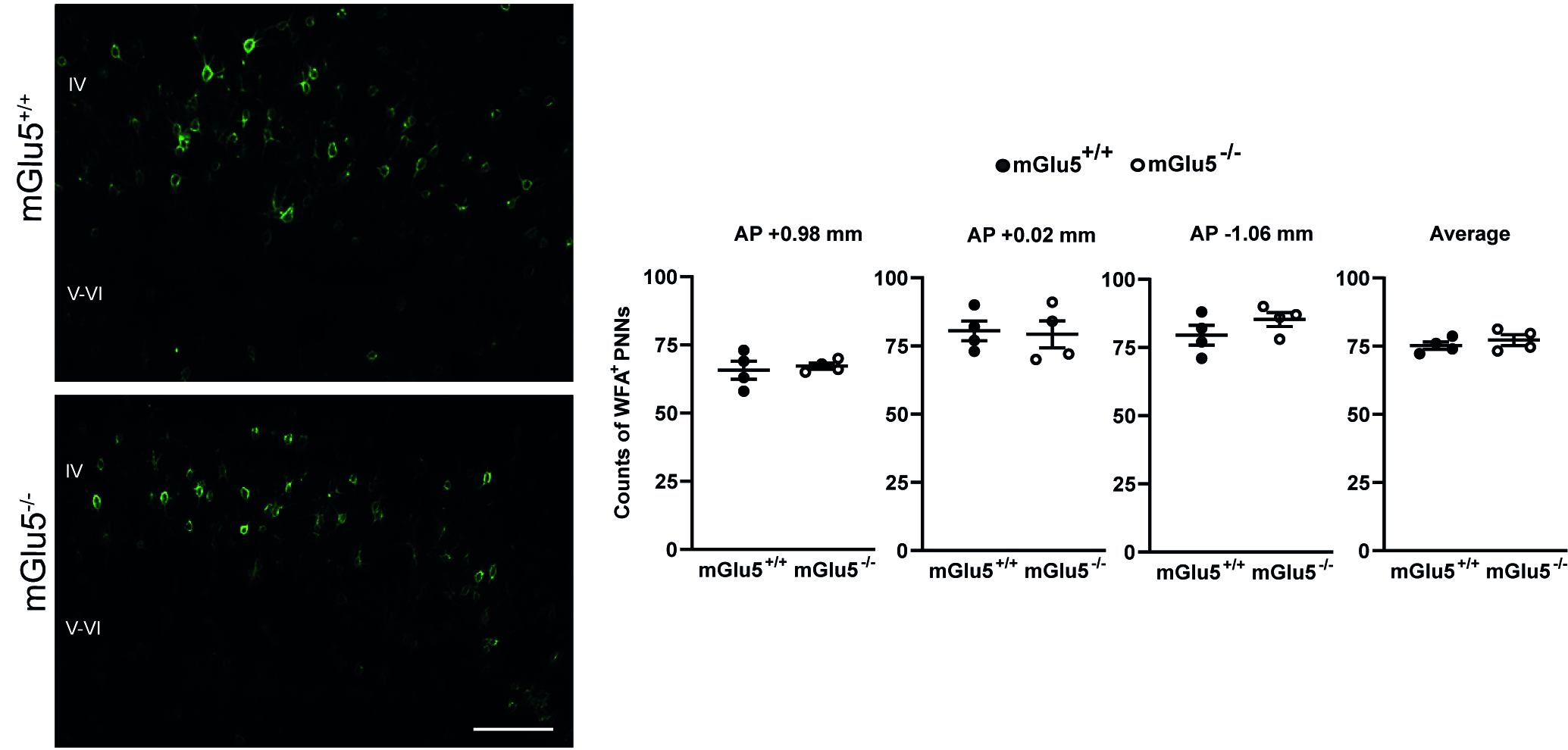

Supplement: Supplementary file 2 — Supplementary Figure 1 [file 41398_2021_1210_MOESM2_ESM.tif]

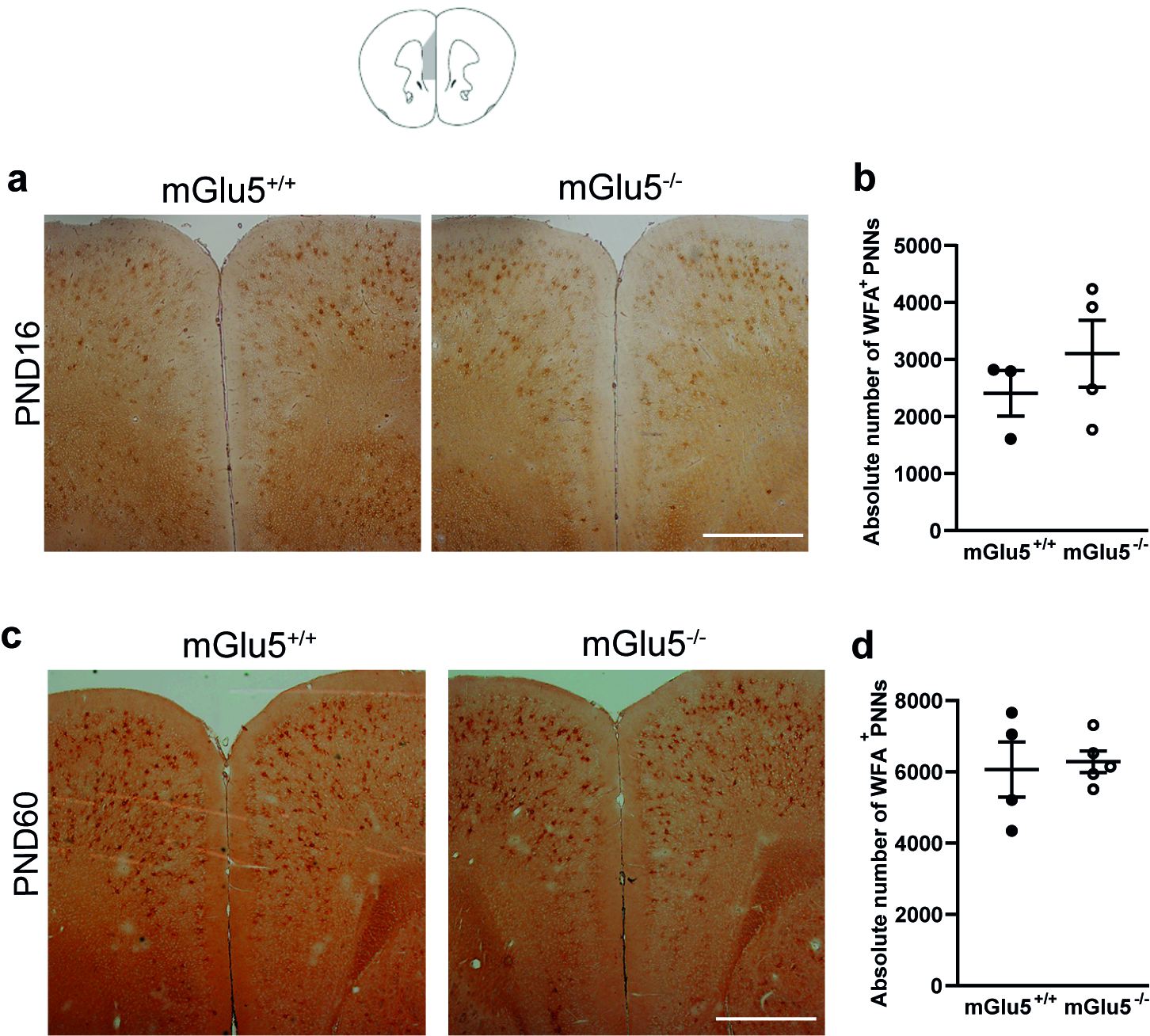

Supplement: Supplementary file 3 — Supplementary Figure 2 [file 41398_2021_1210_MOESM3_ESM.tif]

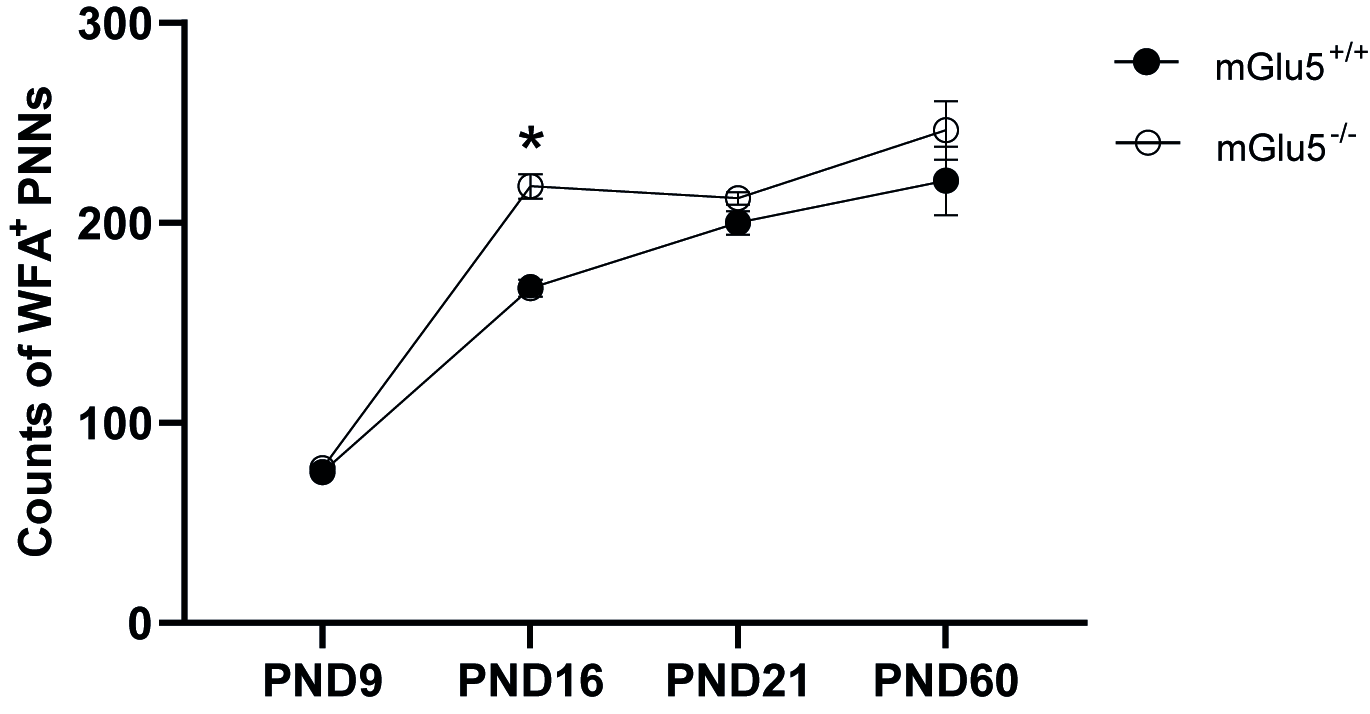

Supplement: Supplementary file 4 — Supplementary Figure 3 [file 41398_2021_1210_MOESM4_ESM.tif]
